# Oral Commissure Ulcer in an Extremely Preterm Infant: A Complication of Endotracheal Tube Fixation at Contralateral Oral Commissure During Lateral Positioning

**DOI:** 10.7759/cureus.75009

**Published:** 2024-12-03

**Authors:** Shintaro Fusagawa, Takuro Sakai, Lisa Igarashi, Takeshi Nishida, Kaori Miki

**Affiliations:** 1 Department of Pediatrics, Sapporo Medical University School of Medicine, Sapporo, JPN

**Keywords:** endotracheal tube fixation, lateral positioning, lip ulcer, mechanical ventilation complication, neonatal intensive care, oral commissure ulcer, preterm infant, pulmonary interstitial emphysema, selective bronchial intubation, shear stress

## Abstract

Lip ulcers associated with endotracheal tube fixation are a known complication in adults, but their prevalence in neonates and preterm infants remains unclear. We report a case of a right oral commissure ulcer that developed during endotracheal tube fixation at the right oral commissure and left lateral decubitus positioning in an extremely preterm infant with unilateral pulmonary interstitial emphysema (PIE).

A male infant was born at 24 weeks and four days of gestation, weighing 696 gm. He required mechanical ventilation for respiratory distress syndrome from birth. On day 66 of life, left lateral positioning and selective right main bronchial intubation were initiated to manage left-sided PIE. The endotracheal tube had initially been fixed at the center or left oral commissure, but on day 101 of life, it was switched to right oral commissure fixation. Eleven days later, an ulcer was observed at the right oral commissure. The ulcer was sutured under local anesthesia. The infant was extubated on day 122 of life and discharged on day 180 of life. The ulcer healed with minimal scarring.

This case suggests that combining endotracheal tube fixation at the oral commissure with contralateral positioning may increase the risk of ulcer formation due to opposing forces from gravity and fixation, resulting in shear stress. Additionally, restricted positioning and continuous sedation may delay the early detection of ulcers. When using lateral positioning, we recommend avoiding contralateral oral commissure fixation of endotracheal tubes and securing the tube to the ipsilateral commissure or upper lip instead.

## Introduction

Extremely preterm infants often require endotracheal intubation and prolonged mechanical ventilation after birth due to pulmonary immaturity [1]. Complications of endotracheal intubation include acute injuries such as vocal cord damage and chronic complications like subglottic stenosis [2]. Additionally, prolonged oral intubation has been associated with palatal grooves and dental defects [3]. Lip ulcers related to the fixation of oral endotracheal tubes have been reported in adult studies with an incidence of 2.6% to 7.3%, indicating they are not rare complications [4]. However, only a few case reports exist regarding neonates and preterm infants, and the actual prevalence remains unclear [5-7].

We report a novel case of oral commissure ulceration that developed during the concurrent management of endotracheal tube fixation at the right oral commissure and left lateral positioning in an extremely preterm infant with unilateral pulmonary interstitial emphysema (PIE). No previous reports have documented oral commissure complications under these conditions. We present this case to alert clinicians that endotracheal tube fixation at the oral commissure combined with contralateral positioning may cause commissure ulceration.

## Case presentation

**Figure 1:**
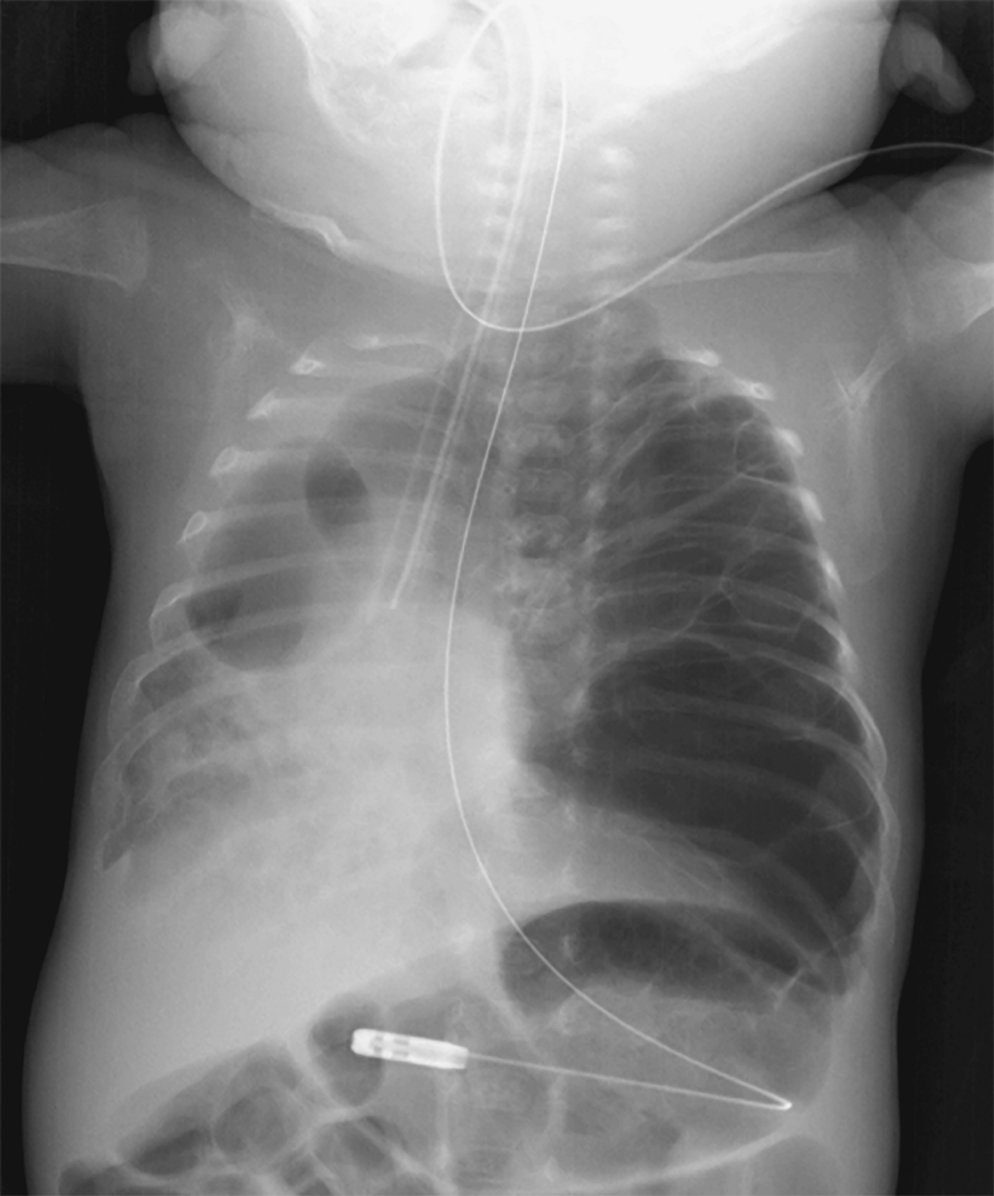
Chest radiograph showing selective right main bronchial intubation for left-sided pulmonary interstitial emphysema (PIE). The endotracheal tube tip is positioned in the right main bronchus to minimize ventilation to the left lung. The left lung shows severe emphysematous changes with multiple large cystic lesions, causing significant rightward mediastinal shift.

The patient was a male infant born at 24 weeks and four days of gestation, weighing 696 gm. Immediately after birth, he underwent endotracheal intubation, intratracheal surfactant administration, and mechanical ventilation for respiratory distress syndrome. Left-sided PIE developed on the second day of life and progressively worsened. As the condition deteriorated, treatments, including high-frequency oscillatory ventilation (HFOV) and steroid administration, were initiated. However, as the PIE continued to worsen, left lateral positioning and selective right main bronchial intubation were initiated on day 66 of life (Figure 1).

Following our institutional standard protocol, the endotracheal tube was initially secured at the midline of the upper lip using elastic adhesive tape. Slight body movements caused tube displacement and respiratory deterioration, requiring continuous sedation. The PIE showed improvement, leading to successful extubation on day 78 of life. However, following the recurrence of the condition, selective right main bronchial intubation was reinstituted on day 92 of life. At this time, unlike the previous midline fixation, the endotracheal tube was secured at the left oral commissure following bronchoscopic confirmation of optimal tip position. Due to frequent tape contamination from saliva accumulation, the fixation site of the endotracheal tube was changed to the right oral commissure on day 101 of life.

**Figure 2:**
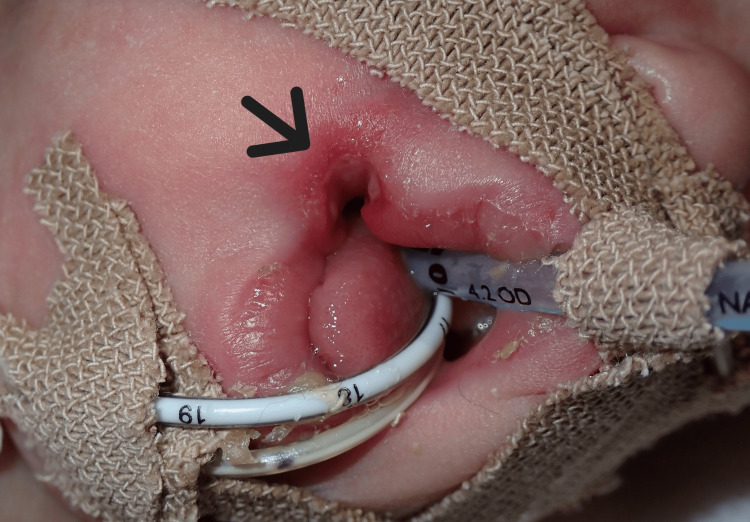
Oral commissure ulcer. The arrow indicates the ulcer at the right oral commissure. The endotracheal tube fixation was relocated from the oral commissure to the upper lip to prevent further tissue damage.

Subsequently, on day 112 of life, 11 days after changing the endotracheal tube fixation site, an ulcer was observed at the right oral commissure (Figure 2).

**Figure 3:**
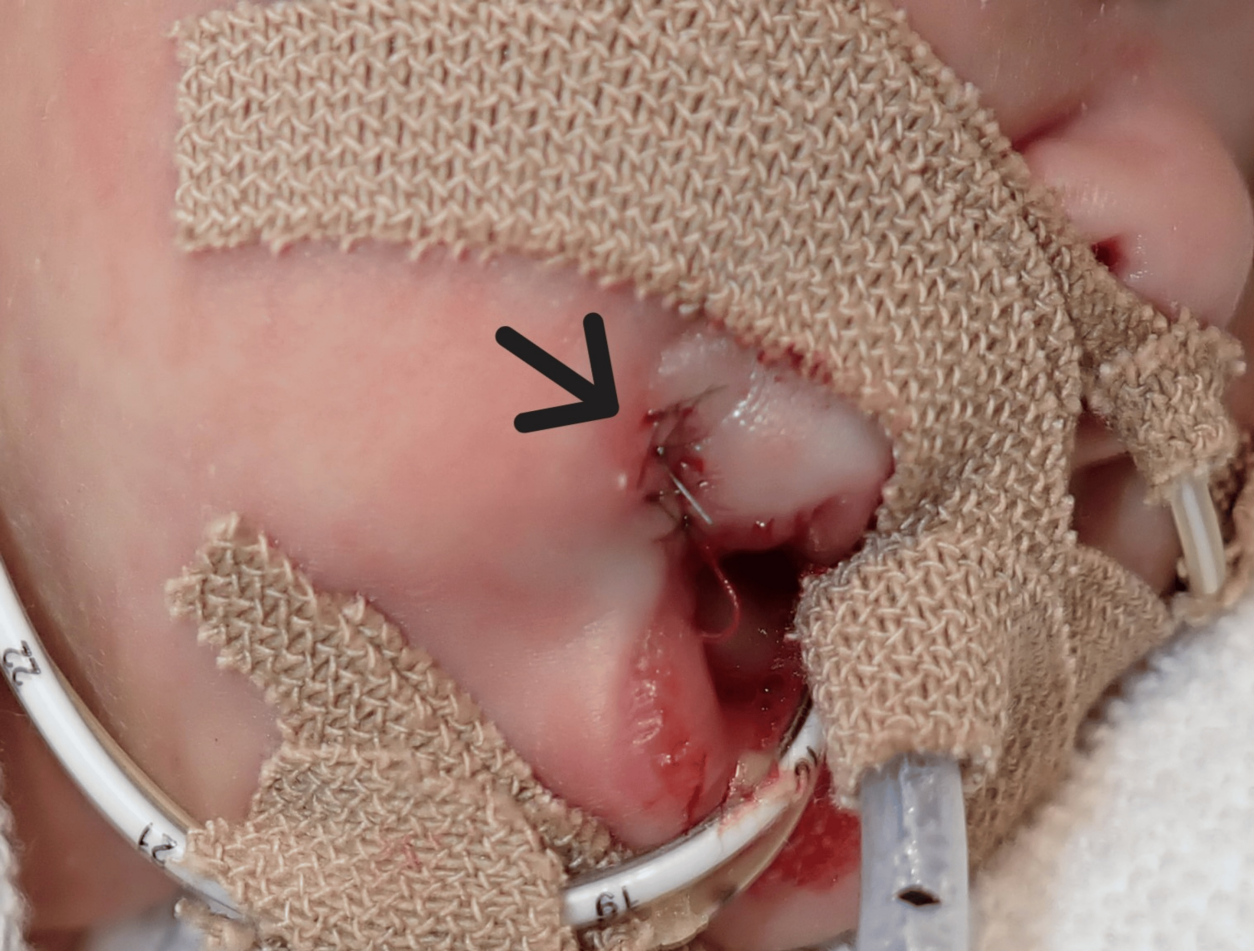
Post suturing of oral comissure ulcer. The black arrow indicates the site sutured under local anesthesia.

Considering that conservative treatment might result in noticeable scarring, the ulcer was sutured under local anesthesia on the same day (Figure 3).

**Figure 4:**
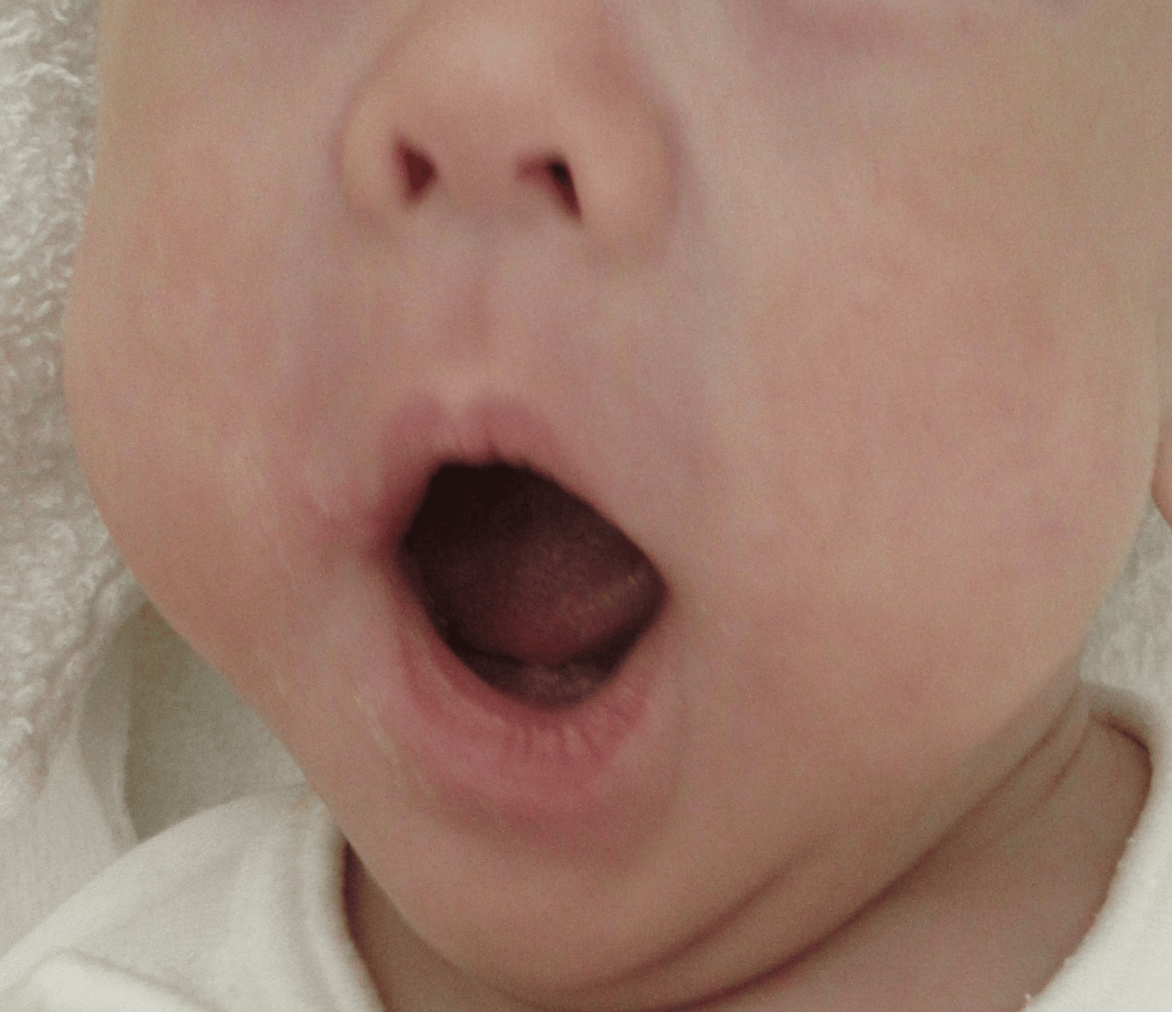
Resolution of oral commissure ulcer with minimal scarring.

After waiting for the PIE to improve, extubation was performed on day 122 of life. Thereafter, his respiratory condition stabilized, and he was discharged on day 180 of life. The ulcer healed with minimal scarring at discharge (Figure 4).

## Discussion

This case demonstrates two findings which are endotracheal tube fixation at the oral commissure with lateral positioning risks ulceration, and restricted movement during selective bronchial intubation may increase ulcer risk and delay detection.

In our patient, prolonged selective right main bronchial intubation and left lateral positioning were necessary to treat the left-sided PIE which is characterized by air leakage from the alveoli into the pulmonary interstitium, can compress the mediastinum and the contralateral lung when unilateral, potentially causing severe respiratory and circulatory disturbances [8]. Lateral positioning therapy and selective bronchial intubation are considered effective non-invasive management methods for unilateral PIE [9,10].

In the left lateral position with endotracheal tube fixation at the right oral commissure, tissues at the right oral commissure are subjected to gravitational pull towards the left side while the tube presses against the skin, creating an upward vertical force. Tape fixation adds additional force. This combination of forces may have generated shear forces on the skin, which has been reported as a major cause of pressure ulcers [11]. Fujioka et al. reported similar lip ulcers caused by shear forces in very low birth weight infants [6]. Furthermore, although the patient was sedated, slight body movements persisted, and when these movements caused the head to tilt leftward, the mechanical pressure exerted by the endotracheal tube against the skin might have been increased. Regarding the time to ulcer development, previous reports documented ulcer onset between 28 and 72 days (median 48 days) [5,6]. While these reports included descriptions of tube fixation sites and patient positioning, none reported cases involving the concurrent use of oral commissure fixation and contralateral positioning. While Makimoto et al. reported cases of early ulcer development associated with Aspergillus infection, our patient showed no evidence of fungal infection [7]. In our case, ulcer formation occurred just 11 days after switching to endotracheal tube fixation at the right oral commissure, presumably due to excessive pressure generated by the combination of contralateral oral commissure fixation and left lateral positioning.

Based on our clinical experience with this case, we propose avoiding endotracheal tube fixation at the oral commissure opposite to the lateral position during lateral positioning management. Fixing the endotracheal tube to the upper lip or the ipsilateral oral commissure may reduce the risk, as gravitational vertical forces would not be generated. In fact, in this case, no significant skin damage was observed during the 78 days of midline fixation and the 9 days of ipsilateral commissure fixation. Additionally, Kahn et al. [5] recommend placing the tube fixation away from the oral commissure, which is considered an effective preventive measure against ulcer formation. However, there are reports of lip ulcers caused by shear forces from fixation tape even with midline fixation [6] and claims that there is no safe place for intraoral fixation [12]. Therefore, complications should be considered regardless of the fixation site. Not only the fixation site but also the fixation method needs to be carefully selected. Studies in adults have reported reductions in tube dislodgement and skin injuries with fixation devices [4]. A commercially available neonatal endotracheal tube holder (NeoBar®, Neotech Products, Valencia, CA, USA) can secure the tube without contacting the lips or oral commissure. We attempted the NeoBar but discontinued it due to respiratory deterioration presumed to be caused by anterior-posterior tube movement and inadvertent left bronchial intubation. A previous study showed that NeoBar did not decrease the rate of unplanned extubation in neonates compared to traditional tape fixation methods [13]. Therefore, while fixation devices may reduce skin injury in neonates, their use needs to be balanced against the risk of tube dislodgement.

Secondly, in clinical conditions requiring restricted movement, such as selective bronchial intubation for unilateral PIE, the risk of ulcer formation may be increased and its detection potentially delayed. This risk is especially relevant when considering positioning strategies. In neonates, prone positioning has been shown to be more effective than supine positioning in stabilizing oxygen saturation and heart rate [14], and it is recommended to avoid prolonged supine positioning and to perform position changes every 3-4 hours [15]. Regular position changes can help distribute pressure points and may contribute to ulcer prevention. However, in our case, therapeutic considerations necessitated maintaining left lateral positioning to compress the left PIE with gravity and restrict airflow to the left lung. Maintaining left lateral positioning for most of the time caused continuous tube pressure on the same area of the oral commissure. This situation was further complicated by challenges in tape management and sedation. While there are no established recommendations for the interval of endotracheal tube tape replacement, neonates typically require frequent tape changes due to loosening from body movements and salivation. However, in our case, due to the severe PIE, even slight tube displacement during tape replacement led to respiratory deterioration, making us reluctant to perform tape changes. Reduced tape replacement limited opportunities for skin assessment, potentially delaying damage detection. Furthermore, although long-term sedation is avoided in preterm infants due to neurological concerns, it was necessary to prevent fatal respiratory deterioration from tube displacement [16]. As a result, the continuous sedation may have masked stress signals from the infant that would typically indicate pressure-related injury, potentially contributing to delayed ulcer detection.

## Conclusions

This case highlights that combining endotracheal tube fixation at the oral commissure with lateral positioning may increase the risk of oral commissure ulceration. Additional factors, such as limited position changes and continuous sedation, might have also contributed to ulcer development in this extremely preterm infant with unilateral PIE. For patients with multiple risk factors, close monitoring is crucial. This includes regular assessment of the endotracheal tube fixation site and careful management of tape replacements. A key consideration is balancing the need for stable endotracheal tube positioning with the prevention of pressure-related complications. Future research should focus on developing optimal endotracheal tube fixation strategies and specialized devices for preterm infants requiring prolonged intubation and lateral positioning.

## References

[1] Sant'Anna G, Shalish W (2024). Weaning from mechanical ventilation and assessment of extubation readiness. Semin Perinatol.

[2] Zubi ZB, Abdullah AF, Helmi MA, Hasan TH, Ramli N, Ali AA, Mohamed MA (2023). Indications, measurements, and complications of ten essential neonatal procedures. Int J Pediatr.

[3] Angelos GM, Smith DR, Jorgenson R, Sweeney EA (1989). Oral complications associated with neonatal oral tracheal intubation: a critical review. Pediatr Dent.

[4] Landsperger JS, Byram JM, Lloyd BD, Rice TW (2019). The effect of adhesive tape versus endotracheal tube fastener in critically ill adults: the endotracheal tube securement (ETTS) randomized controlled trial. Crit Care.

[5] Kahn DJ, Spinazzola R (2005). Acquired oral commissure defect: a complication of prolonged endotracheal intubation. J Perinatol.

[6] Fujioka M, Oka K, Kitamura R, Yakabe A (2008). Upper lip pressure ulcers in very low birth weight infants due to fixation of the endotracheal tube. J Neonatal Nurs.

[7] Makimoto M, Kawasaki Y, Inomata S, Tamura K, Yoshida T (2017). Early upper lip pressure ulcer in a preterm neonate. Pediatr Int.

[8] Sahota RJ, Anjum F (2022). Pulmonary interstitial emphysema. In: StatPearls [Internet].

[9] Al-Mudares F, Fernandes CJ (2021). Unilateral neonatal pulmonary interstitial emphysema managed conservatively: A case report. Pediatr Pulmonol.

[10] Bowman ED, Murton LJ (1984). Selective intubation in pulmonary interstitial emphysema: experience in five patients. Aust Paediatr J.

[11] Hoogendoorn I, Reenalda J, Koopman BF, Rietman JS (2017). The effect of pressure and shear on tissue viability of human skin in relation to the development of pressure ulcers: a systematic review. J Tissue Viability.

[12] Amrani G, Gefen A (2020). Which endotracheal tube location minimises the device-related pressure ulcer risk: The centre or a corner of the mouth?. Int Wound J.

[13] K Loganathan P, Nair V, Vine M, Kostecky L, Kowal D, Soraisham A (2017). Quality improvement study on new endotracheal tube securing device (NeoBar) in neonates. Indian J Pediatr.

[14] Elsagh A, Lotfi R, Amiri S, Gooya HH (2019). Comparison of massage and prone position on heart rate and blood oxygen saturation level in preterm neonates hospitalized in neonatal intensive care unit: A randomized controlled trial. Iran J Nurs Midwifery Res.

[15] Parry S, Ranson P, Tandy S (2023). Positioning and Handling Guideline. https://www.neonatalnetwork.co.uk/nwnodn/wp-content/uploads/2023/11/Positioning-and-Handling-Guideline-2023-Final-1.pdf.

[16] Moser JJ, Archer DP, Walker AM, Rice TK, Dewey D, Lodha AK, McAllister DL (2023). Association of sedation and anesthesia on cognitive outcomes in very premature infants: a retrospective observational study. Can J Anaesth.

